# Highly Sensitive Immunoresistive Sensor for Point-Of-Care Screening for COVID-19

**DOI:** 10.3390/bios12030149

**Published:** 2022-02-28

**Authors:** Tianyi Li, Scott D. Soelberg, Zachary Taylor, Vigneshwar Sakthivelpathi, Clement E. Furlong, Jong-Hoon Kim, Sang-gyeun Ahn, Peter D. Han, Lea M. Starita, Jia Zhu, Jae-Hyun Chung

**Affiliations:** 1 Department of Mechanical Engineering, University of Washington, Seattle, WA 98195, USA; lit24@uw.edu (T.L.); zntaylor@uw.edu (Z.T.); viggysak@uw.edu (V.S.); 2 Departments of Medicine, Division of Medical Genetics and Genome Sciences, University of Washington, Seattle, WA 98195, USA; scottjs@uw.edu (S.D.S.); clem@uw.edu (C.E.F.); 3 School of Engineering and Computer Science, Washington State University, Vancouver, WA 98686, USA; jh.kim@wsu.edu; 4 Industrial Design, University of Washington, Seattle, WA 98195, USA; ahnsang@uw.edu; 5 Department of Genome Sciences, University of Washington, Seattle, WA 98195, USA; petedhan@uw.edu (P.D.H.); lstarita@uw.edu (L.M.S.); 6 Brotman Baty Institute for Precision Medicine, Seattle, WA 98195, USA; 7 Department of Laboratory Medicine and Pathology, University of Washington, and Vaccine and Infectious Disease Division, Fred Hutchinson Cancer Research Center, Seattle, WA 98195, USA; jiazhu@uw.edu

**Keywords:** single-walled carbon nanotubes, immunoresistive sensor, resistance ratio, COVID-19, point-of-care diagnosis

## Abstract

Current point-of-care (POC) screening of Coronavirus disease 2019 (COVID-19) requires further improvements to achieve highly sensitive, rapid, and inexpensive detection. Here we describe an immunoresistive sensor on a polyethylene terephthalate (PET) film for simple, inexpensive, and highly sensitive COVID-19 screening. The sensor is composed of single-walled carbon nanotubes (SWCNTs) functionalized with monoclonal antibodies that bind to the spike protein of SARS-CoV-2. Silver electrodes are silkscreen-printed on SWCNTs to reduce contact resistance. We determine the SARS-CoV-2 status via the resistance ratio of control- and SARS-CoV-2 sensor electrodes. A combined measurement of two adjacent sensors enhances the sensitivity and specificity of the detection protocol. The lower limit of detection (LLD) of the SWCNT assay is 350 genome equivalents/mL. The developed SWCNT sensor shows 100% sensitivity and 90% specificity in clinical sample testing. Further, our device adds benefits of a small form factor, simple operation, low power requirement, and low assay cost. This highly sensitive film sensor will facilitate rapid COVID-19 screening and expedite the development of POC screening platforms.

## 1. Introduction

The emerging SARS-CoV-2 virus, including its reemerging coronavirus variants, continues to pose a serious threat to global public health and economy. As of 3 January 2022, the World Health Organization (WHO) reported worldwide 281,808,270 confirmed cases, including a total of 5,411,759 deaths due to SARS-CoV-2 infection caused Coronavirus Disease 2019 (COVID-19) [1]. The novel coronavirus has impacted industry, economy, and many facets of our daily life. Currently, COVID-19 continues to spread rapidly in communities around the world.

The diagnosis of COVID 19 is based on respiratory system assessment, nucleic acid-based method, and immunoassays. Respiratory system assessment, such as chest CT and wearable breath monitoring, lacks specificity [2,3]. Alternatively, respiratory rate, oxygen saturation, and heart rate are used for symptom management [4]. For an accurate diagnosis, nucleic acid amplification tests (NAATs) such as quantitative reverse transcription-polymerase chain reaction (qRT-PCR) assay [5,6] are considered the gold standard for COVID-19 screening [7]. These methods are currently used for screening patients for COVID-19 infection. However, these commercial assays require 4~6 h of assay time, as well as expensive, bulky equipment and skilled technicians [8]. More recently, a loop-mediated isothermal amplification (LAMP) assay has been developed to accelerate the screening. By combining a shorter screening time with a more convenient detection tool such as colorimetry, a POC NAAT detection assay could be developed. For example, a colorimetric reverse-transcriptional LAMP assay was developed for the detection of SARS-CoV-2 [9].

Many rapid tests have been developed to detect whole virus, viral RNA, immunoglobulin from infection, and antigens without nucleic acid amplification [10]. For example, the volatile organic compounds (VOCs) of exhaled gas could be detected for screening [11]. Among these, many commercially available rapid tests detect antigens. For example, lateral flow immunoassays (LFIA) rely on fluorescent, colorimetric, or electrochemical detection methods to detect the presence of SARS-CoV-2 viral proteins. Such antigen tests are being used as pre-screening or home screening tools under the Emergency Use Authorization (EUA) [12]. These devices are simple, fast, and low cost but are limited to sensitivity. The BinaxNOW™ COVID-19 Antigen Self Test was reported to have a lower limit of detection (LLD) of 10^4^ copies/mL, which is 100-fold less sensitive than qRT-PCR tests [13]. Other Ag-based commercial POC antigen tests have a limit of detection (LOD) greater than 10^6^ copies/mL [14]. These reported levels are not sensitive enough to detect the target virus in nasal swab samples at an early stage of infection where the viral load can be lower than 100 copies/mL, according to a clinical study [15]. Immunoglobulin tests detecting Immunoglobulin G (IgG) or Immunoglobulin M (IgM) lack specificity, do not respond to early-stage infection, and may produce false positives for the vaccinated population [16,17,18]. Other detection methods are more promising in accuracy and screening time. Some of them are antibody-coated field-effect transistors [19,20], paper-based electrochemical sensors [21], nanoparticles (NP) based electrochemical sensors [22], optical sensors [23], surface plasmon resonance (SPR) [24], and surface-enhanced Raman spectroscopy (SERS) [25].

Single-walled carbon nanotubes (SWCNTs) are one of the potential candidates for simple and inexpensive detection with high sensitivity and specificity. Resistive SWCNT sensors can detect target binding by electrostatic interaction or work function modification [26,27,28,29]. Viral particles and bacteria can be detected by monitoring this resistance change. Using similar technology, the LLD of the swine influenza virus (H1N1) was 177 TCID50 (50% tissue culture infective dose)/mL [30]. SWCNTs functionalized with heparin detected dengue virus at concentrations as low as 840 TCID50/mL [31]. For detecting H1N1, the LLD was 1 plaque-forming unit (PFU)/mL [32]. In our previous report, *Mycobacterium tuberculosis* (MTB) was detected at 100 CFU/mL in sputum samples using SWCNT sensors combined with magnetic particles [33]. However, this assay did not demonstrate sufficient sensitivity for detecting early-stage SARS-CoV-2 patients. Also, because the sensor substrate was made on silicon chips, the fabrication and integration costs were too high for an inexpensive screening assay.

In this paper, we constructed a resistive SWCNT biosensor on a polyethylene terephthalate (PET) film for low-cost COVID-19 screening. An array of silver electrodes were silkscreen-printed on SWCNTs for large-scale production. The sensitivity and specificity were characterized for SARS-CoV-2 in phosphate-buffered saline (PBS) and nasal swabs. The relative resistance ratio of both control and sensor was measured upon binding of the spike protein of SARS-CoV-2. The SWCNT sensor also detected the virus in positive nasal swabs previously screened with qRT-PCR. The presented biosensor will facilitate the development of a POC COVID-19 rapid screening platform that has high sensitivity, low assay cost, and low power requirements.

## 2. Materials and Methods

### 2.1. Sensor Configuration and Operation

**Figure 1:**
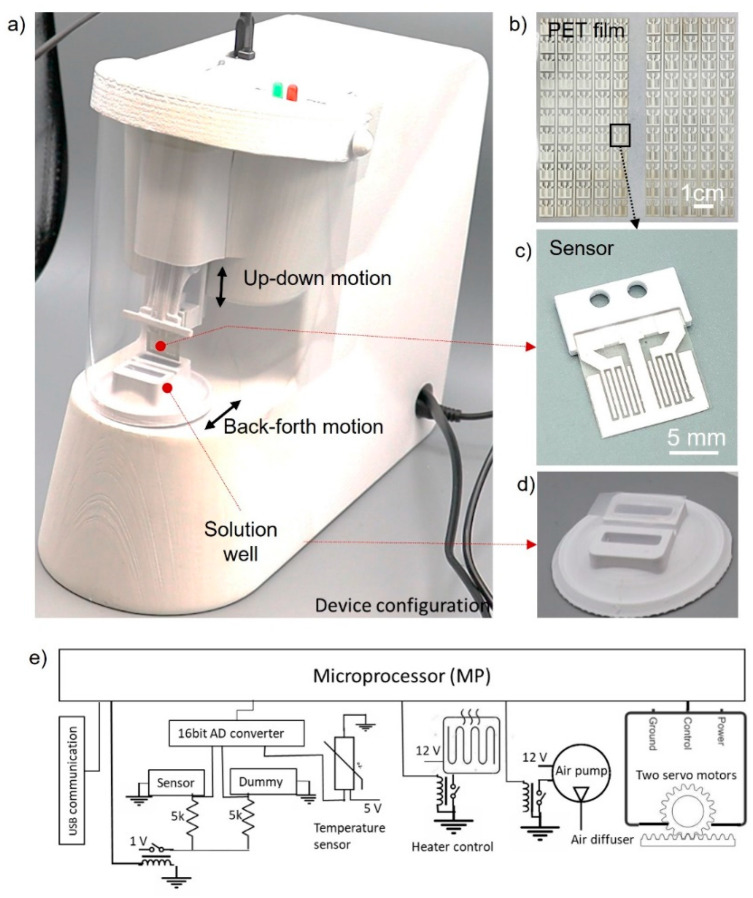
(**a**) Analyzer for rapid COVID-19 screening. (**b**) Sensors are made on a PET film coated with single-walled carbon nanotubes immobilized with antibodies. (**c**) A sensor consists of a SARS-CoV-2 sensor and a control sensor. (**d**) Sample cup containing 1×PBS with 1mL-target analyte and 1.1 mL-DI water. (**e**) Configuration of the electric circuit. USB communication unit, resistance measurement units, temperature sensing unit, heating and control unit, air blow with a diffuser, and two servo motors for vertical and horizontal movements.

The SWCNT immunosensor was designed to handle a minimally processed nasal swab sample suspended in 1 mL of PBS, yielding a ‘Yes or No’ answer. The prototype device (Figure 1a) was composed of two linear motors for vertical sensor movement (dipping, withdrawal, and rinsing) and horizontal sample cup movement (solution change and mixing). Resistance was measured for both sensor and control electrodes. A heater was embedded under a sample cup to maintain a temperature of 36 °C to speed up the antibody-antigen binding and maintain a consistent temperature through the assay steps and between samples.

The SWCNT sensor was fabricated by spin-coating SWCNTs onto a PET film and silkscreening silver electrodes over the SWCNT surface (Figure 1b). A SWCNT sensor was composed of a sensing electrode and a control electrode (Figure 1c). The SWCNTs on the sensing electrode were covalently conjugated with monoclonal antibodies specific to the spike protein of SARS-CoV-2. The SWCNTs on the control electrode element were conjugated with bovine serum albumin (BSA). The interdigitated electrodes offered a large surface area for high sensitivity.

The sample cup had two liquid compartments: one held 1×PBS from a nasal swab sample, and the other held deionized (DI) water for the washing steps (Figure 1d). For immunocomplex formation, a buffered solution (PBS) was needed. Since the electrostatic interaction of SWCNTs with antigen was a key mechanism for detection, the masking effect by ions in the PBS needed to be reduced by rinsing the sensor with DI water. The vertical motion of the sensor played two roles in detection. One was to eliminate nonspecific binding, and the other was to rinse and dry the sensor completely. By carefully controlling the sensor withdrawal step, the capillary and viscous forces removed the nonspecifically bound molecules.

The sensor surface was mostly dried by using a low withdrawal speed (1 mm/s). The remaining water drop at the edge of the sensor surface was removed by an air diffuser designed to blow air uniformly over the sensor surface. An air pump (flow rate: 4 L/min) was connected to the air diffuser, which was powered on only at the withdrawal step. The air pump operated at a low flow rate so that aerosols could not be generated but the sensor surface could completely dry. The resistance measurement confirmed the complete dryness of the sensor surface. A sensor with residual water yielded low and unpredictable resistance readings, while a dry sensor showed stable values. If a sensor remains wet, the silver electrode on the sensor surface could be quickly oxidized due to the potential.

Figure 1e shows the configuration of the control and electrical units, including a microcontroller (Atmega 328p). The resistance measurement units were installed to measure the resistance change of the sensor elements. A joule heating element with a temperature sensor was installed to maintain the temperature between 35 and 37 °C. An air pump was controlled with a relay switch. Two servo motors were combined with a rack and pinion gear to provide accurate linear movements.

The major innovation of the SWCNT sensor was to use the ratio of the resistance change of a sensing electrode in comparison to that of a control electrode. In our previous work [33], the sensing mechanism was characterized. Despite the high sensitivity, the previous sensor was susceptible to temperature change and aging. In this SWCNT sensor, such effects could be calibrated by comparing the resistance change of sensing and control electrodes. Based on the comparison, we could identify the subtle resistance change from the target binding by negating environmental changes.

The use of PET films as sensor substrates significantly reduces the material and manufacturing costs in comparison to silicon chips. However, the rough surface on a PET film made the contact resistance higher. According to our study previously performed using an atomic force microscope, the roughness of the surface ranges from 15 to 80 nm. By patterning silver electrodes on the SWCNT surface, the contact resistance could be stably controlled [33].

**Figure 2:**
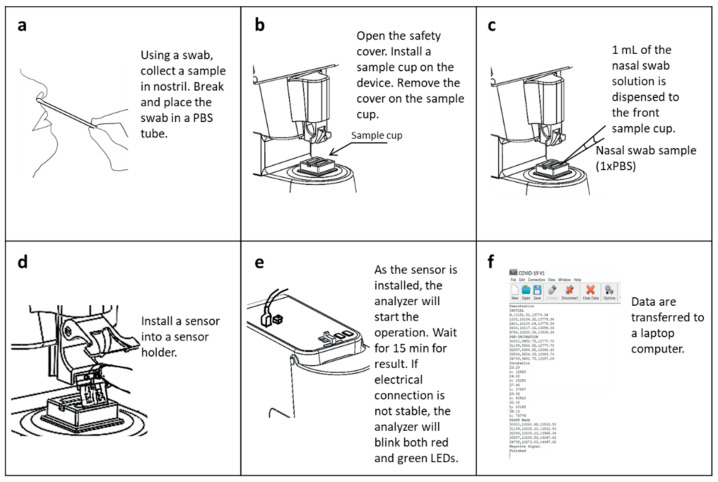
Screening protocol (**a**) Nasal swab sample is collected from nostril. The nasal swab is immersed and eluted in 1×PBS. (**b**) Install a sample cup where 1.1 mL DI water is contained. (**c**) A nasal swab sample of 1 mL is transferred to a sample cup. (**d**) Install a sensor with inspection of electrical connection. (**e**) Press a reset switch to begin detection protocol. (**f**) Data are collected after 15 min.

For the screening protocol, a swab was used to collect a sample from inside the nostrils (Figure 2). The sample swab was then immersed and stirred in 1.2 mL-1×PBS. A sample cup containing 1.1 mL-DI water was installed in the analyzer. One mL of the 1×PBS solution containing the swab sample was then transferred to the sample cup. After sensor installation, the 15 min-screening protocol was initiated. Once the measurement was completed, the data were analyzed and transferred to a laptop computer.

For the screening protocol, the resistances of the sensing and control electrodes were measured five times during the course of one assay. The resistance measurement steps are described with the detection protocol in Table 1. At the initial stage, the resistances of the sensing and control electrodes were measured (R_0_s_ and R_0_c_). Subsequently, the sensor was immersed into the DI water for 10 seconds, withdrawn, and dried with an air pump for 40 s. The resistances were measured after air dry with an average of five readings (R_1_s_ and R_1_c_). The resistance values from DI water showed the initial status of SWCNT sensors. If the ratio (P_1_ = P_1_s_ / P_1_c_) was in the range of 0.9~1.1, the measurement went to the next step. If the ratio was not in the range, the screening was halted due to poor sensor functionality. Once the quality control step passed, the sensor was then dipped into the sample cup containing the nasal swab sample in 1 mL of 1×PBS. The sensor was agitated in this sample at a speed of 1mm/second back and forth for 10 min with the liquid temperature at 36 °C. After a 10 min incubation, the sensor was rinsed in the DI water well at a stirring speed of 2 mm/s for 10 s. The sensor was subsequently air-dried, and the resistance values of R_2_s_ and R_2_c_ were measured. The same dipping rinse steps were repeated without stirring twice, during which a set of (R_3_s_, R_3_c_) and (R_4_s_, R_4_c_) was measured.

Regarding the quality control step, the initial resistance measurement was used to find the functionalization quality of a sensor in comparison to control. It was found that the errors were caused by the activation and deactivation step of the carboxyl group on the SWCNT surface. The errors could occur due to small differences in the manual fabrication steps, including pipetting and incubation steps. In our test, the fabrication yield was ~70%. In the future, quality control needs to be improved to increase the fabrication yield.

### 2.2. Sensor Fabrication

**Figure 3:**
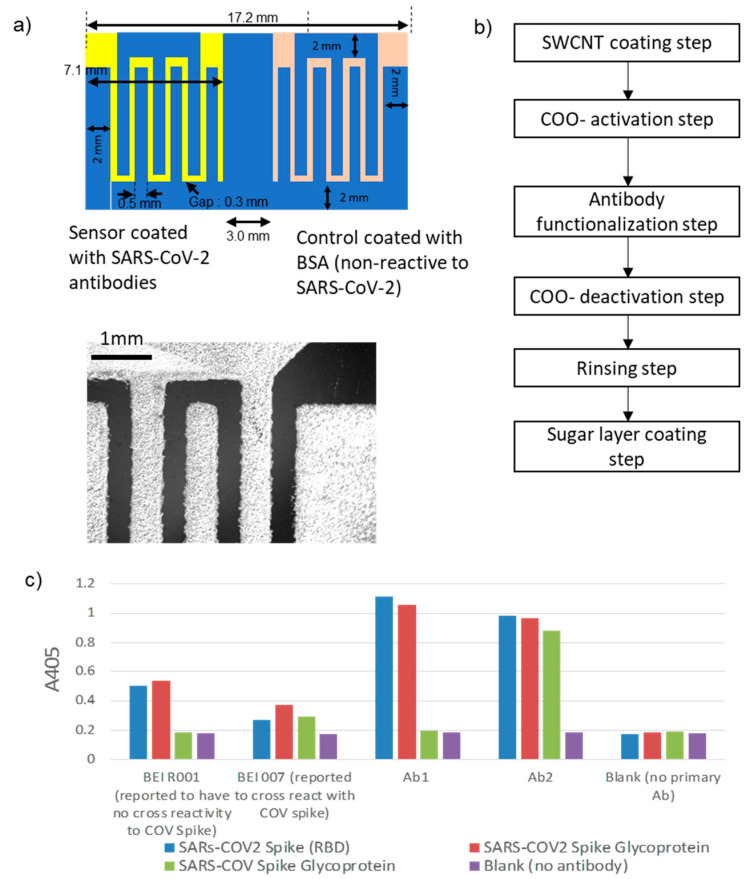
(**a**) Sensor configuration. Sensor electrodes are coated with SARS-CoV-2 antibodies. Control electrodes are with BSA. (**b**) Antibody functionalization step on SWCNTs. (**c**) Specificity test results to SARS-CoV and SARS-CoV-2 of antibodies using ELISA.

An SWCNT sensor was prepared by screen-printing silver electrodes on an SWCNT-coated polyethylene terephthalate (PET) substrate (Figure 3a). Polyethyleneimine solution (0.1% PEI in DI water) was prepared by diluting a stock solution (50%, Millipore-Sigma, St. Louis, MI, USA). The diluted PEI solution was spin-coated on a 100 μm-thick PET film (3M Highland 903) at 3000 rpm for 3 min. SWCNTs were spread on the PET surface (200 × 200 mm^2^). Using a radius of 100 mm, the relative centrifugal field (RCF) was 1006*g*. The spin-coater we used was VTC-50A (MTI Corporation, Richmond, CA, USA). The PEI-coated film was cured at 100 °C for 10 min. Carboxylic acid-functionalized SWCNTs (SWCNT-COOH, Millipore-Sigma) were dispersed in double-distilled water (ddH_2_O, Millipore-Sigma) at 0.3 mg/mL. Using a horn-type sonicator, SWCNTs were dispersed for 20 min. The SWCNT suspension was spin-coated on a PEI-coated PET film at 3000 rpm for 3 min, followed by curing at 100 °C for 10 min. Silver ink was used to silkscreen electrodes onto the SWCNT-coated sensor surface. The silver we used was AG-510 conductive ink (Kayaku Advanced Materials, Westborough, MA, USA). The screen printer was the LS-34 (New Long, Japan). And the screen-printing mask was fabricated by Sefar Inc. (Depew, NY, USA). After patterning, the silver ink was cured at 120 °C for 15 min. The sensor was composed of two resistive sensing sections; the left section detected the SARS-CoV-2 virus, and the right section served as a control electrode. Both sections contain two interdigitated electrodes whose fingers were separated by 0.3 mm (Figure 3a).

To covalently immobilize antibodies onto the SWCNT-COOH, a protocol was modified from the reference [34] to activate carboxyls on the SWCNT for covalently bonding to amino groups on antibodies (Figure 3b). A solution of 38.5 mg/mL EDC (Thermo #22980) and 11 mg/mL S-NHS (Thermo #PG8-2071) in DI water was prepared. 60 µL of this solution was pipetted onto each side of the sensor and incubated for 15 min at room temperature. Sensors were then washed with DI water from a wash bottle and dried with a stream of air from a compressor. A 20 µg/mL solution of either virus-specific antibody or BSA was then added ( left side = 80 µL Ab, right side = 80 µL BSA ) and incubated at room temperature for 2 h. Sensors were then rinsed with DI water and dried with a stream of air from a compressor. To quench any remaining amine-reactive groups, the pH was raised to 8.0 to speed hydrolysis. This was done by adding 300 µL PBS (pH 8) to cover both sides of each sensor and incubating overnight at room temperature. Sensors were then rinsed with DI water and dried with a stream of air from a compressor. A layer of a trehalose and dextran mixture was added to protect the antibody surface during storage. Each sensor was dipped in a trehalose/dextran solution [2.5% trehalose, 2.5% dextran (average MW 500,000)] [35] to cover the lower 2/3 of the sensing area. Sensors were then cured for 2 H in a 37 °C incubator. In the following tests, all the sensors were stored at room temperature and used within one week.

### 2.3. Antibody Characterization

We compared four commercial antibodies for binding to spike protein of both SARS-CoV and SARS-CoV-2. Antibody cross-reactivity was measured to SARS-CoV-2 whole spike protein (BEI NR-52308), SARS-CoV-2 receptor-binding domain (RBD) section of spike protein (Sino Biological 40592-V05H), and SARS coronavirus whole spike (S) Protein (BEI NR-722).

For this experiment, two antibodies were previously tested for cross-reactivity with both SARS-CoV and SARS-CoV-2 spike protein (both whole and RBD). Sino Biological 40150-R007 has been previously shown to be specific to the SARS-CoV-2 spike S1 domain and spike receptor-binding domain (RBD) and has also shown to be cross-reactive with the SARS-CoV Spike S1 domain and RBD. Sino Biological 40150-R001 was specific to the SARS-CoV-2 spike protein RBD as shown previously in ELISA, with cross-reactivity to the SARS-CoV-2 spike S1 protein. However, cross-reactivity was not observed in ELISA with S1 glycoproteins from SARS-CoV.

Protein binding plates (Immulon 2HB, ThermoFisher Scientific 3455) were coated with 100 µL of a 2 µg/mL antigen (spike protein) solution for 24 H at room temperature. Following antigen binding, plates were washed w/DPBS from a wash bottle and then blocked with a 1 mg/mL BSA solution in DPBS (200 µL) and incubated for 30 min at 37 °C. After washing excess BSA from the plate with DPBS from a wash bottle, a solution of the primary antibody (100 µL of 1 µg/mL in DPBS) was added and incubated for 30 min at 37 °C. The plate was then washed with DPBS, and a 100 µL of anti-rabbit conjugated HRP (Invitrogen 31460) at a 1:2000 dilution was added and incubated at 37 °C. After 30 min, the excess secondary antibody was removed by washing with DPBS, then 100 µL ABTS substrate (ThermoFisher 37615) was added and incubated for 10 min at room temperature. The plate was then read for absorbance on a microplate reader at A405nm.

The ELISA results showed the specific binding of BEI R001 and Ab1 to SARS-CoV-2 whole spike and RBD, while Ab2 shows additional cross-reactivity to SARS-CoV whole spike protein (Figure 3c).

### 2.4. Sensor Characterization

The sensor resistance change was characterized for sensors with and without antibodies. According to our observations, the functionalization step to activate and deactivate carboxyl groups on the SWCNT surface dominated the sensor resistance change. After the carboxyl groups were activated, the sensor resistance change was not consistent. By comparing the resistance change of sensors with and without antibodies, deactivation steps could be modified to result in a predictable change of SWCNT resistances. The resistance values for each step were measured as shown in Table 1. By considering the resistance change of the sensor without antibodies as a control, the functionalization protocol for the sensors with antibodies was optimized. The resistance change was compared for each step. For the comparison tests, 1mL of 1×PBS was used as the target solution with 1.1 mL of DI water. In addition, an initial test was conducted to study the resistance ratio change for 1×PBS, and 1×PBS spiked with SARS-CoV-2 (1000 genome equivalents/mL).

### 2.5. Sensitivity Tests

To test the sensitivity, various concentrations of inactivated SARS-CoV-2 [BEI #NR-52287 (Irradiated, Novel Coronavirus, 2019-nCoV/USA-WA1/2020)] were suspended in PBS buffer. The SARS-CoV-2 source consisted of the cell lysate and supernatant from Cercopithecus aethiops kidney epithelial cells infected with SARS-CoV-2. The virus was gamma-irradiated for inactivation. The initial concentration from BEI resources was 1.7 × 10^9^ genome equivalents/mL. The initial stock solution was serially diluted to 10 particles/mL by serial 10 fold dilutions. A 1 mL solution of the prepared virus sample was loaded into a sample cup. After the initial resistance measurement, an SWCNT sensor was dipped in DI water, followed by air-drying and the 2nd resistance measurement. The SWCNT sensor was then immersed in 1 mL of a virus solution for 10 min with an agitation (3 mm/s), followed by an air-dry and the first washing. Two more dipping, air drying, and washing steps were repeated to measure all resistance changes. Based on the initial resistance value, all the normalized resistance values were calculated for data processing. For the control experiments, 1×PBS buffer without target virus was used.

### 2.6. Test Using Nasal Swab Samples

To evaluate the lower limit of detection for SARS-CoV-2, nasal swab samples were collected from deidentified healthy volunteers. After the complete drying of swabs for a few hours in air, the swab samples were immersed in 1 mL PBS for 1 min with gentle stirring. Subsequently, 500 µL of the target analyte (SARS-CoV-2) in PBS was mixed with 500 µL of the eluted swab solution. The 1 mL solution was used to test the LLD. The spiked concentrations of SARS-CoV-2 ranged from 10^2^ to 10^5^ genome equivalents/mL in steps of 10-fold dilutions. The resistance values were measured and processed as previously described.

Additional sensitivity tests were conducted for the SARS-CoV-2 concentrations of 100, 250, 500, and 1000 genome equivalents/mL in order to estimate LOD. Based on the results, a linear analysis was conducted to estimate the accurate detection limit.

### 2.7. Cross-Reactivity Test

For cross-reactivity study, the response for SARS-CoV-2 (10^3^ genome equivalents/mL) was compared with Staphylococcus Epidermidis (S. Epi at 10^3^ CFU/mL), Mycobacterium Tuberculosis (MTB at 10^3^ CFU/mL), and Staphylococcus Aureus (SA) at 10^3^ CFU/mL, respiratory syncytial virus (RSV, 10^6^ genome equivalents/mL) and influenza A (H1N1, 10^6^ genome equivalents/mL). The nontargeted samples were suspended in 1×PBS, which was mixed with nasal swab samples. Each sample was repeated three times (N = 3).

### 2.8. Test Using Clinical Samples

To validate the assay performance, 12 positive and 10 negative patient samples were tested from previously determined RT-qPCR assayed samples. The samples were collected by anterior nares swabs for the Husky Coronavirus Testing research study (IRB) that provided testing to faculty, staff, and students at the University of Washington in Seattle, WA, USA (PMID: 34805425). Dry samples were transported and eluted in 1 mL Tris-EDTA (PMID: 34286830) and stored at −80 °C. Among the samples, 12 positive and 10 negative samples were randomly chosen and deidentified for sensor testing. The positive samples included alpha and delta variants of SARS-CoV-2. The sample collection and testing procedure were approved by the institutional review board (IRB) at the University of Washington (Husky Testing number: STUDY00011148).

Since the collected sample volume after RT-qPCR assays was only 100 µL, the sample was diluted to 1 mL using 1×PBS. The 1mL samples were tested by the prepared SWCNT sensors in a non-blinded fashion. RT-qPCR Ct values were determined after thawing and dilution to serve as a direct comparator to the SWCNT.

The clinical testing lab was approved for SARS-CoV-2 screening assay based on RT-qPCR [36]. The Northwest Genomics Center (NWGC) SwabDirect SARS-CoV-2 detection assay was validated for the workflow of COVID-19 screening. The sensitivity, specificity, accuracy, and precision of an extraction-free protocol were validated through the evaluation of the contrived positive specimens along with known positive and negative clinical specimens.

## 3. Results and Discussion

### 3.1. Sensor Characterization

When the sensor was fabricated without antibodies, the average resistance values were 9.88 ± 1.51 kΩ (N = 12). When the antibodies were immobilized on the sensor surface, the average resistance values increased to 24.5 ± 2.69 kΩ. The normalized difference between the sensor and control resistances was 0.037 ± 0.010 for the sensors immobilized without antibodies (N = 6). The normalized difference between the sensor and control resistances was −0.057 ± 0.061 for the sensors with immobilized antibodies (N = 6). Before antibody immobilization, the normalized resistance difference between sensing and control electrodes was positive. After antibody immobilization, the normalized resistance difference changed to a negative value due to the difference between SARS-CoV-2 antibodies and BSA on the electrodes.

**Figure 4:**
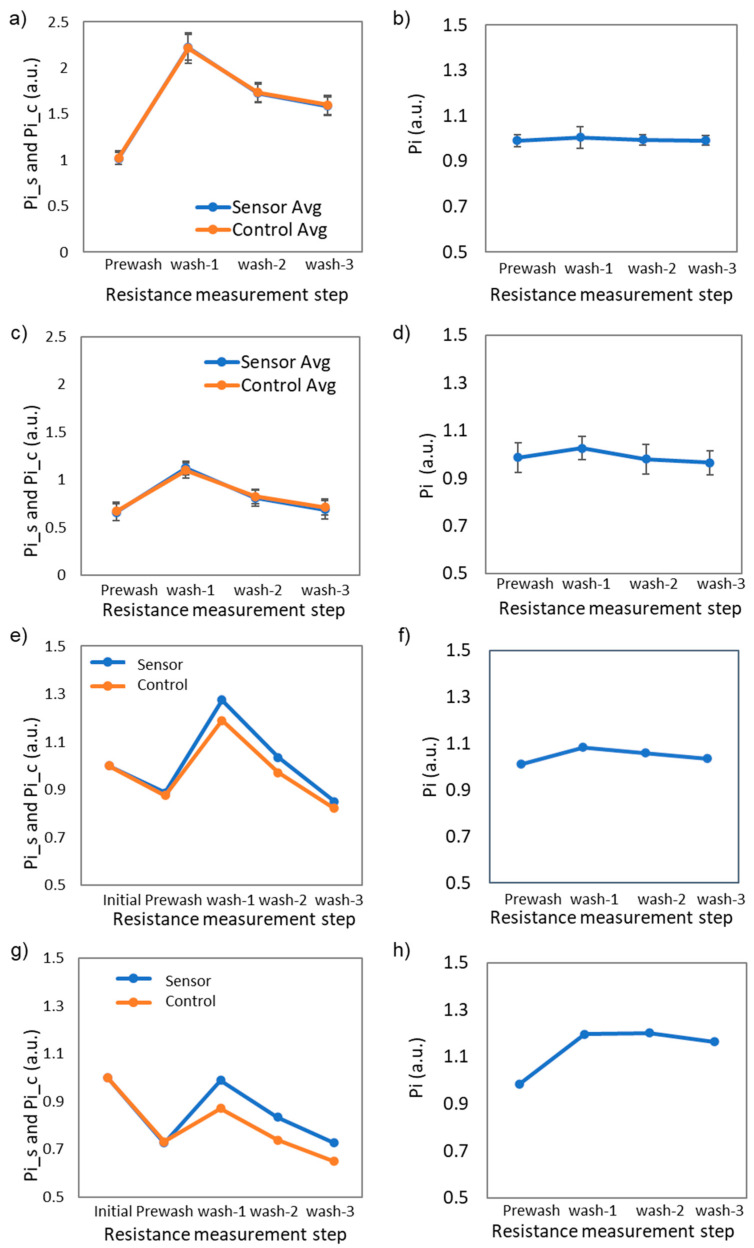
(**a**) P_i_s_ and P_i_c_ changes for the sensors without antibodies (N = 6). (**b**) P_i_ changes for the sensors without antibodies (N = 6). (**c**) P_i_s_ and P_i_c_ changes for the sensors with antibodies (N = 6). (**d**) P_i_ changes for the sensors with antibodies (N = 6). (**e**) P_i_s_ and P_i_c_ changes for the antibody-immobilized sensors for a negative nasal swab sample. (**f**) P_i_ changes for the antibody-immobilized sensors for a negative nasal swab sample. (**g**) P_i_s_ and P_i_c_ changes for the antibody-immobilized sensors for a negative nasal swab sample spiked with SARS-CoV-2 (10^3^ genome equivalents/mL). (**h**) P_i_ changes for the antibody-immobilized sensors for a negative nasal swab sample spiked with SARS-CoV-2 (10^3^ genome equivalents/mL).

To study the resistance change for the sensors with and without antibodies, both sensors were tested by the analyzer using the screening protocol (Figure 4). For this experiment, 1×PBS was used without any target analytes. As described in Table 1, the initial resistance values (R_0_) were collected from the sensor (R_0_s_) and control (R_0_c_) electrodes. The initial resistance values served as a baseline for the following measurements. Figure 4a,b show P_i_s_ and P_i_c_ value changes and their ratio change P_i_ (i = 0, 1, 2, 3, and 4 ) for the sensors without antibodies. The normalized resistances of P_i_s_ and P_i_c_ were close to 1 at the prewash step in DI water, followed by an increase at the first rinsing step. The increase of the normalized resistance values was caused by the ion adsorption on the surface when the sensor was immersed in 1×PBS. The normalized resistance values decreased at the second and third rinsing steps as the ions were depleted in DI water.

Figure 4c,d present the P_i_s_ and P_i_c_, and P_i_ value changes with antibodies, respectively. With immobilization of antibodies, the prewash values of P_1_s_ and P_1_c_ reduced to 0.67, but the overall trend was similar to the sensors without antibodies. The P_i_ value showing the relative change ratio between the sensing and control electrodes showed an increased response at the first wash. The P_3_ and P_4_ values corresponding to the second and third rinsing showed a trend converging to 1. According to the initial characterization, the trend for the P_i_ values with and without antibodies approached 1 with multiple rinsing steps.

Table 2 shows one example of the measured resistance values for sensing and control electrodes. The resistance values were converted to the resistance ratio in Figure 4c. If the sensor resistance was greater than 40 kOhm, the sensor was not used due to an unpredictable sensitivity. If the resistance was lower than 10 kOhm, the SWCNTs were too abundant to achieve adequate sensitivity.

An example of the resistance change ratios for the negative control in a nasal swab sample is shown in Figure 4e,f. The P_i_s_ and P_i_c_ values for sensing and control electrodes appeared to diverge more than pure PBS solution (Figure 4e), but the P_i_ values between sensor and control remained close to 1 (Figure 4f). Figure 4h are examples of the resistance changes for the positive control of 10^3^ genome equivalents/mL in nasal swab samples. When the target viruses were captured on the sensor surface, the P_i_s_ and P_i_c_ became larger at the rinsing steps (Figure 4g). The P_i_ values clearly showed the difference between sensing and control electrodes in Figure 4h. In comparison to the negative control signal in Figure 4f, the positive control signal in Figure 4h showed the normalized value >1 due to the resistance change difference for sensing and control electrodes.

### 3.2. Signal Processing and Sensitivity Tests

**Figure 5:**
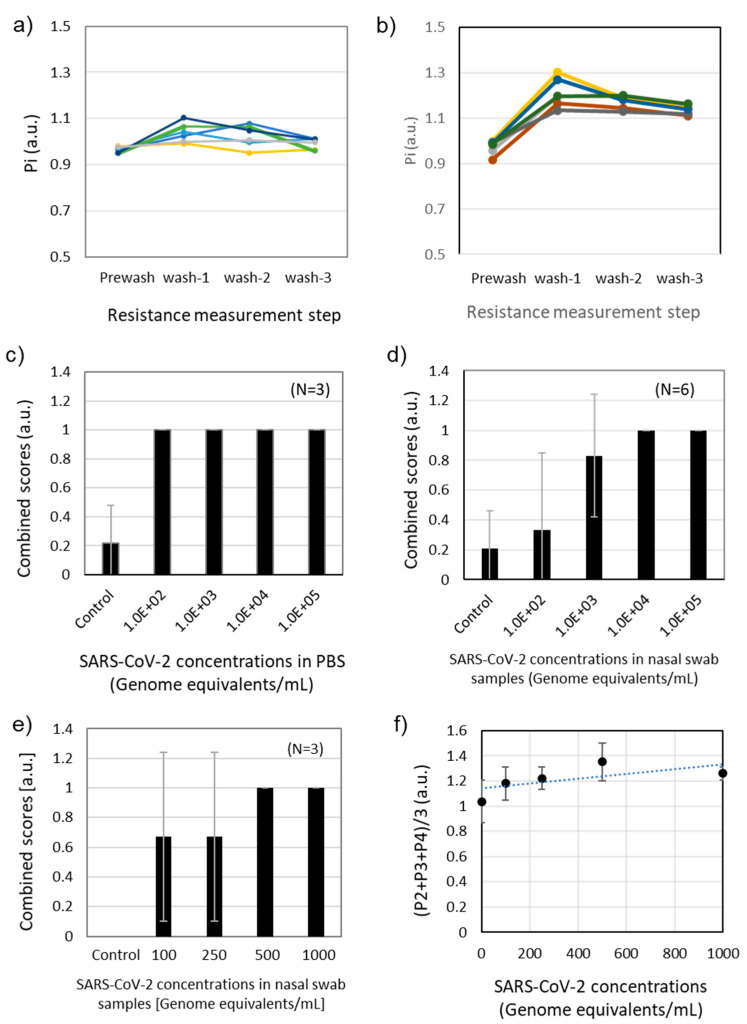
(**a**) Pi changes for negative nasal swab samples (N = 6). (**b**) Pi changes for nasal swab samples spiked with SARS-CoV-2 (1000 genome equivalents/mL). (N = 6) (**c**) Combined scores for various concentrations of SARS-CoV-2 in 1×PBS (N = 3). Negative control (N = 9) (**d**) Combined scores for various concentrations of SARS-CoV-2 in nasal swab samples (N = 6 for positive samples, N = 12 for negative controls). (**e**) Dose response tests for estimating the limit of detection in nasal swab samples spiked with SARS-CoV-2. The concentrations of SARS-CoV-2 are 0, 100, 250, 500, and 1000 genome equivalents/mL (N = 3). (**f**) Average values of P2, P3, and P4 for the SARS-CoV-2 at the concentrations of 0, 100, 250, 500, and 1000 genome equivalents/mL in nasal swab samples (N = 3).

Before the dose-response tests in nasal swab samples, the P_i_ value changes were monitored for negative and positive nasal swab samples to determine the signal processing methods for screening. Figure 5a,b show the P_i_ values for negative swab samples and positive swab samples spiked with 10^3^ genome equivalents/mL-SARS-CoV-2 (N = 6), respectively. Overall, the P_2_, P_3_, and P_4_ values of the positive samples were greater than those of negative samples. However, the P_i_ values were not clearly differentiated in certain cases, which was attributed to sensor production batches and potential errors in washing steps. It was interesting to find that the positive signals showed a larger slope of P_i_ between prewash (P_1_) and the first wash (P_2_). Also, the positive signals showed a lower average slope of (P_2_ – P_3_) and (P_3_ – P_4_). According to the results, we defined two parameters to determine the positive screening results as described in the following conditions.

If (P_2_ – P_1_) > 0.12, a score of C_1_ = 0.5 is given. (1)

If (P_4_ – P_1_) > 0.1, a score of C_2_ = 0.5 is given. (2)

If the combined score of C_1_ + C_2_ was equal to 1, it was positive. If the combined scores were 0 or 0.5, it was negative.

Using the combined scores, the dose response tests of SARS-CoV-2 in PBS were conducted. Figure 5c shows the combined scores for SARS-CoV-2 in PBS at the concentrations of 10^2^ and 10^5^ genome equivalents/mL. All the signals of the positive samples showed the combined value of 1. When the nasal swab samples spiked with 10^2^ and 10^5^ genome equivalents/mL were used, two out of six samples showed the combined value of 1 at 10^2^ genome equivalents/mL, and five out of six samples showed the combined value of 1 at 10^3^ genome equivalents/mL (Figure 5d).

To estimate the LLD in nasal swab samples, further testing was conducted for the SARS-CoV-2 concentrations of 100, 250, 500, and 1000 genome equivalents/mL (Figure 5e). At concentrations of 100 and 250, only one sensor out of three showed the combined score of 1. The combined scores at 500 and 1000 genome equivalents/mL were 1 (N = 3). When the average values of P_2_, P_3_, and P_4_ were used, the linear increase of the resistance ratio was observed (Figure 5f). Based on the linear approximation of the average P_i_ values, the LOD was 350 genome equivalents/mL. The LOD of 350 genome equivalents/mL was better than typical nucleic amplification-based assays being used for COVID-19 screening. Given that swab samples were replete with human cell fragments, bacteria, and other interferents, these results also demonstrated the specificity of the developed SWCNT sensors.

The average value at 1000 genome equivalents/mL in Figure 5f was a little lower than those of other concentrations, which could be caused by the different fabrication batches. Among the dataset, all the sensors except those used for 1000 genome equivalents/mL were from the same batch. Note that the P_2_ value was the parameter that was conventionally used for screening using SWCNT sensors. Due to the batch-to-batch variation, the analog value of the resistance ratio could not be used directly. Instead, the combined scores were better for determining the positive and negative screening results.

### 3.3. Cross-Reactivity Test

**Figure 6:**
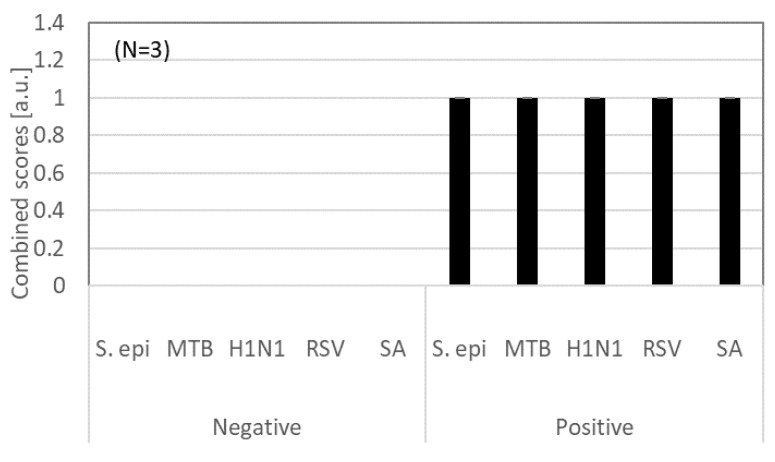
Combined scores for negative and positive nasal swab samples spiked with S. epidermidis, MTB, H1N1, RSV, and SA. The concentration of SARS-CoV-2 was 10^3^ genome equivalents/mL. The bacterial concentrations of *S. epidermidis*, MTB, and *S.a* were 10^6^ CFU/mL. The viral concentrations of H1N1 and RSV were 10^8^ genome equivalents mL (N = 3).

Figure 6 shows the testing of cross-reactivity of the SWCNTs sensors. The nasal swab samples spiked with *S. epidermidis, MTB, H1N1, RSV*, and *S.a.* were used for negative samples and showed no response. The positive samples were nasal swab samples spiked with 10^3^ genome equivalents/mL-SARS-CoV-2, S. *epidermidis, MTB, H1N1, RSV*, and *SA.* The combined scores clearly showed the difference between negative and positive samples.

### 3.4. Clinical Sample Test Results 

Clinical testing was performed on previously frozen SARS-CoV2 positive and negative samples collected in TE buffer and tested with RT-PCR. Since only 100 µL of the samples were available in TE buffer, the positive and negative samples were diluted 10-fold using 1×PBS. The PCR test results showed that the positive samples included alpha and delta variants. Among the twelve positive and ten negative samples, an SWCNT sensor showed one false positive result for a negative sample. According to the results, the clinical sensitivity was 100%, and the clinical specificity was 90% (Table 3).

## 4. Conclusions

**Figure 7:**
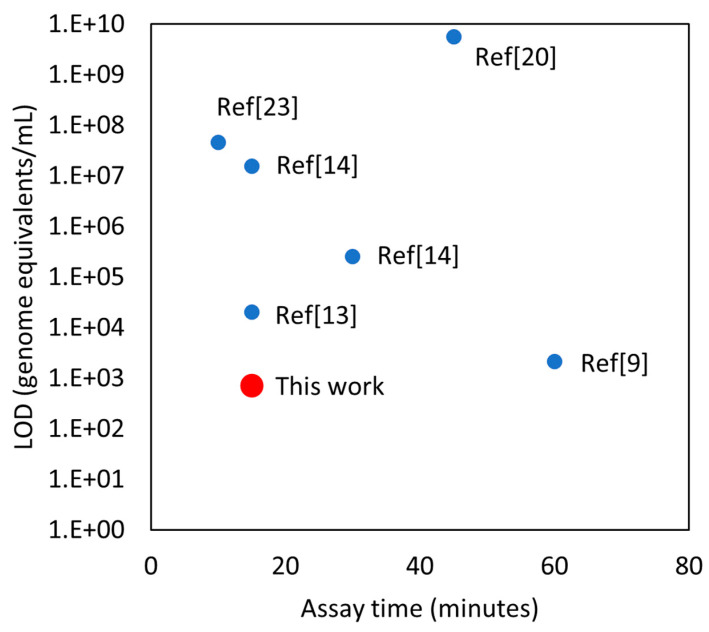
Comparison between this sensor and prior reports in terms of assay time and limit of detection (LOD). Several assumptions have been made to unify the units of LOD. Each virus contains 2 RNA strands. 1 PFU/mL is assumed to be 10,000 RNA copies/mL [37]. One virus is assumed to have 24 Spike Glycoprotein (S) and each of them has a mass of 100 kDa [38]. In reference [7], the reported time is the response time instead of the assay time.

In summary, an immunoresistive SWCNT sensor was developed to specifically detect SARS-CoV-2 in nasal swab samples. The analytical LOD was 350 genome equivalents/mL with a detection time of 15 min. The analytical LOD was better than point-of-care screening nucleic acid detection assays. In comparison to other antigen detection assays, the detection limit was 2~3 orders of magnitude more sensitive (Figure 7). To achieve such high sensitivity and specificity, the relative resistance change of an SWCNT sensor was measured in comparison to a control sensor. To improve the clinical sensitivity and specificity, a combined score using two parameters based on the resistance ratio was used. According to clinical sample tests, the assay showed 100% sensitivity and 90% specificity. The SWCNT sensors detected both alpha and delta variants. The simple resistive measurement will allow rapid screening by minimally trained personnel. Also, a minimal power requirement (<1 W) will be important for point-of-care (POC) screening in limited-resource settings.

Regarding the analyzer and assay cost, the analyzer was constructed using mass-produced parts to reduce the manufacturing cost. The assay cost was very low due to the plastic film patterned with silkscreened silver electrodes and antibodies. The fabrication yield of the SWCNT sensors was ~70%. The quality control process needs to be improved for a higher fabrication yield. Currently, we are developing a larger scale production of sensors with a quality management system included.

## Figures and Tables

**Table 1:** Screening protocol, resistance measurement, parameters, and screening time.

Resistance Measurement Step	Resistance Values and Ratios	Parameters	Time (minutes) Total Time = 15 min
Before testing	P_0_s_ = R_0_s_/ R_0_s_; P_0_c_ = R_0_c_/ R_0_c_	P_0_ = P_0_s_/ P_0_c_ = 1	0.5
Prewash in DI water	P_1_s_ = R_1_s_/ R_0_s_; P_1_c_ = R_1_c_/ R_0_c_	P_1_ = P_1_s_/ P_1_c_	1
Incubation	Not Measured	None	10
Wash 1	P_2_s_ = R_2_s_/ R_0_s_; P_2_c_ = R_2_c_/ R_0_c_	P_2_ = P_2_s_/ P_2_c_	1
Wash 2	P_3_s_ = R_3_s_/ R_0_s_; P_3_c_ = R_3_c_/ R_0_c_	P_3_ = P_3_s_/ P_3_c_	1
Wash 3	P_4_s_ = R_4_s_/ R_0_s_; P_4_c_ = R_4_c_/ R_0_c_	P_4_ = P_4_s_/ P_4_c_	1

**Table 2:** Resistance values for negative control shown in Figure 4c.

	Sensor (kΩ)	Control (kΩ)
Initial resistance	23.368	24.048
pre-wash	13.140	13.115
wash 1	24.239	23.551
wash 2	16.822	17.075
wash 3	13.518	14.379

**Table 3:** Comparison of PCR results and SWCNT sensor results for positive and negative clinical samples (N/A means Ct > 40).

Positive	Reference PCR		* SWCNT Sensor	Negative	Reference PCR	* SWCNT Sensor
Sample ID	Ct Value	Variants		Sample ID	Ct Value (>40)	
XXXX61b	22.5	alpha	Positive	XXXX8ba7	N/A	Negative
XXXXa4dc	33	alpha	Positive	XXXX4fbd	N/A	Negative
XXXXf041	23.5	alpha	Positive	XXXX6ea9	N/A	Negative
XXXX89d7	30	alpha	Positive	XXXXa467	N/A	Negative
XXXX996	29.4	alpha	Positive	XXXXd9ee	N/A	Negative
XXXXa320	38.5	alpha	Positive	XXXX297e	N/A	Negative
XXXX1b27	31.3	delta	Positive	XXXX4907	N/A	** Positive
XXXXf10e	30.6	delta	Positive	XXXXde69	N/A	Negative
XXXX8c9c	26.5	delta	Positive	XXXX07e3	N/A	Negative
XXXXf06c	31.2	alpha	Positive	XXXX4d9f	N/A	Negative
XXXX5456	30.4	delta	Positive			
XXXX76fb	23.5	alpha	Positive			

* Unblinded trial. ** A PCR negative sample shows the strong positive signal of the SWCNT sensor. The sample collection and testing procedure has been approved by the institutional review board (IRB) at the University of Washington (Husky Testing number: STUDY00011148). The sample IDs are deidentified with XXXX.

## Data Availability

Not available.
